# Theoretical Study on the Sensing Mechanism of Novel Hydrazine Sensor TAPHP and Its ESIPT and ICT Processes

**DOI:** 10.3389/fchem.2019.00932

**Published:** 2020-01-15

**Authors:** Songsong Liu, Jiajun Lu, Qi Lu, Jianzhong Fan, Lili Lin, Chuankui Wang, Yuzhi Song

**Affiliations:** Shandong Province Key Laboratory of Medical Physics and Image Processing Technology, School of Physics and Electronics, Shandong Normal University, Jinan, China

**Keywords:** ESIPT, hydrogen bond, ICT, IGM, topology analysis, CVB index, sensing

## Abstract

The photophysical and photochemical properties of the novel hydrazine sensor TAPHP and the TAPDP generated by the cyclization reaction of TAPHP with hydrazine are investigated using the density functional theory and time-dependent density functional theory. The results show that both the excited-state intramolecular proton transfer and intramolecular charge transfer can occur for TAPHP and TAPDP. Analysis of bond parameters and infrared vibrational spectra indicate that hydrogen bonds are enhanced in the first excited state, which is beneficial to excited-state intramolecular proton transfer. The strength of hydrogen bonds is also visualized by using the independent gradient model and topological analysis. The core-valence bifurcation index and bond critical point parameters are further employed to measure hydrogen bonds. The reaction path of proton transfer is obtained through the potential energy curves. The excitation of TAPHP and TAPDP is attributed to the charge transfer excitation, which is determined by the characteristics of the hole-electron distribution. The reaction site and product configuration are verified by atomic charge and ^1^H-NMR spectra. The negative free energy difference indicates that the reaction between TAPHP and hydrazine can proceed spontaneously. In addition, the absorption and fluorescence spectra agree well with the experimental results, confirming that TAPHP is an excellent sensor of hydrazine.

## Introduction

Hydrogen bond (HB) observed in DNA, water, proteins, and other materials (Zhao and Han, 2011; Tanioku et al., 2013; Gole et al., 2014; Ling and Gutowski, 2016; Huang et al., 2018) formed between a hydrogen (H) atom of one molecular fragment D-H and another atom A (i.e., D-H^**…**^A) (Li et al., 2011; Wilcken et al., 2013; An et al., 2016, 2017; Ma et al., 2018; Zhao et al., 2019). Where D atom is connected to H atom by a covalent bond (or ionic bond), the A atom has a high electron density and is easy to attract hydrogen proton. HBs are ubiquitous in molecular structures and have been the research focus due to their distinct properties. Studies have shown that many sensing mechanisms can be well-explained by HB interactions, such as the excited-state intramolecular proton transfer (ESIPT), fluorescence quenching, and intramolecular charge transfer (ICT) (Wen and Jiang, 2004; Plasser et al., 2009; Rawat and Biswas, 2014; Zhu et al., 2017). The ESIPT was first found by Weller when the double fluorescence emission of methyl salicylate was observed (Weller, 1956; Zhou et al., 2014). Such a pioneering discovery soon led to a flurry of studies on the proton transfer mechanism. Recently, Han et al. discovered that HB interaction can be enhanced in excited states (Zhao and Han, 2007; Zhao et al., 2007; Chai et al., 2009; Liu et al., 2018; Song et al., 2019). According to Weller, the ESIPT process of methyl salicylate was induced by its intramolecular HB.

ESIPT is a photochemical process that produces a tautomer with a different electronic structure from the original excited form through a four-level photo cycle (Enol-Enol^*^-Keto^*^-Keto) (Mahanta et al., 2011; Demchenko et al., 2013; Ray et al., 2014; Chen et al., 2016; Kumpulainen et al., 2016; Liu et al., 2019b). Among them, the role of H proton in the ESIPT process is extremely important due to its active characteristics. The energy difference between the initial and the relaxed excited state will power the proton transfer. The ESIPT model can be described by a variety of reaction coordinates. One of the simplest ways is to use the D-H bond length as the reaction coordinate. As the D-H bond is stretched, the reaction will continue to drive (Lee et al., 2013). Molecules with ESIPT property have been used in various fields due to their unique photophysical properties (Liu et al., 2019a). One of the more valuable applications is the use of fluorescent sensors for ions, biomolecules, and chemicals (Padalkar and Seki, 2016). For example, Wang et al. synthesized a fluorescence sensor to monitor methanol based on ESIPT characteristics (Qin et al., 2018). Yang et al. proposed that Al^3+^ sensor can be prepared by ESIPT and PET mechanisms (Yue et al., 2017). Interestingly, a novel hydrazine sensor TAPHP was recently synthesized by Wang et al. (Luo et al., 2018).

**Figure 1:**
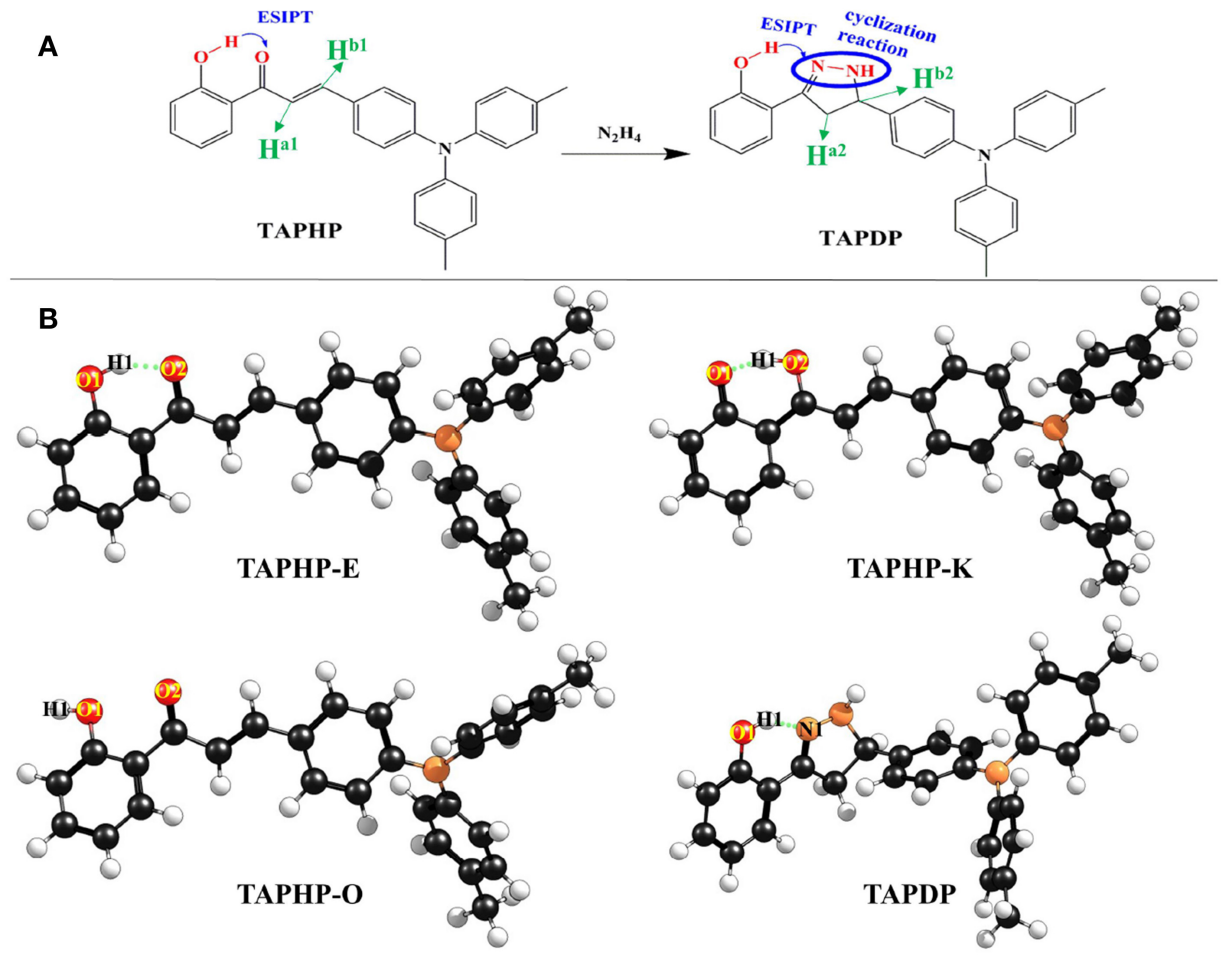
**(A)** Chemical structures of probe TAPHP and TAPDP formed by cyclization reaction between TAPHP and hydrazine. **(B)** Optimized geometries of the TAPHP and TAPDP.

Hydrazine is a colorless oily liquid, which is well-soluble in polar solutions such as water and alcohol. The benefit is that it can make fuel for rockets and jet engines, mirror silver plating and foaming agent. While, the drawback is that it can cause severe skin erosion and damage to the eyes and liver due to its extreme toxicity. Therefore, we are motivated to investigate the sensing mechanism of the newly designed hydrazine sensor TAPHP. It is commendable that the ESIPT process is considered in the probe skeleton, which produces long-wavelength emission to avoid autofluorescence of the probe. We found that the TAPDP molecule generated by the cyclization reaction can also occur ESIPT and ICT, which was not observed experimentally. Hydrazine can be specifically recognized by the probe since its amino group has stronger nucleophilicity than other amines. However, it is difficult to distinguish hydrazine and hydroxylamine composed of -OH group and -NH_2_ group by ordinary fluorescent probes. The probe TAPHP can recognize hydrazine and hydroxylamine because it can undergo cyclization reaction with hydrazine, and the -OH group on hydroxylamine has a lower nucleophilicity, which is not conducive to cyclization reaction. As shown in Figure 1A, the cyclization reaction of TAPHP with hydrazine is accomplished by condensation of an amine with ketone and the conjugation addition of another amine of hydrazine to α, β-unsaturated carbonyl group. In this work, we focused on the ESIPT and ICT processes of TAPHP and TAPDP through analyzing bond lengths and bond angles, infrared (IR) vibration spectra, potential energy curves (PECs), and hole-electron distribution, etc. The calculated atomic charge and proton nuclear magnetic resonance (^1^H-NMR) spectra verify the reaction site. The sensing mechanism of TAPHP is also well-confirmed by the large stokes shift of the fluorescence spectra.

## Theoretical Calculation Methods

All *ab initio* calculations were carried out by using Gaussian 16 suite (Frisch et al., 2016). The density functional theory (DFT) (Lee et al., 1988) and time-dependent density functional theory (TDDFT) (Becke, 1993) methods are adopted to optimize the geometries of TAPHP and TAPDP in the ground (S_0_) and first excited (S_1_) states without any constraints, respectively. A series of functionals [B3LYP (Miehlich et al., 1989), CAM-B3LYP (Yanai et al., 2004), PBE0 (Adamo and Barone, 1999), M06-2X (Zhao and Truhlar, 2008), and PW6B95 (Grimme et al., 2011)] suitable for calculating weak interactions are tested. Table 1 shows that the absorption peaks of TAPHP and TAPDP calculated by functional PW6B95 are the closest to the experimental results. Meanwhile, the triple-zeta valence quality with one set of polarization functions (TZVP) (Eichkorn et al., 1997; Vargas et al., 2000) performs well in this work. Therefore, we employ PW6B95/TZVP as the most reliable method in the current work. In order to be as consistent as possible with the experimental environment, the solvent effect [dimethyl sulfoxide (DMSO)] (Bordwell et al., 1990) based on the polarizable continuum model (PCM) using the integral equation formalism variant (IEFPCM) is utilized in *ab initio* calculations (Cammi and Tomasi, 1995; Cances et al., 1997). To improve calculation accuracy, the implicit solvation model based on density (SMD) (Marenich et al., 2009) was employed to calculate free energy and ^1^H-NMR spectra. The IR vibration analysis after geometric optimization shows that all the molecular configurations are the true minimum. The independent gradient model (IGM), topological analysis, and core-valence bifurcation (CVB) index are employed to visualize the strength of intramolecular HBs as well as electron-hole distribution were obtained by using Multiwfn program (Lu and Chen, 2012a,b). Additionally, to quantitatively describe the reaction path of proton transfer, the PECs are obtained by scanning the bond length of O_1_-H_1_ with a step of 0.1 Å. In the present work, the enol and keto forms of TAPHP and TAPDP are represented as TAPHP-Enol, TAPHP-Keto, TAPHP-Enol^*^, TAPHP-Keto^*^, TAPDP-Enol, TAPDP-Keto, TAPDP-Enol^*^, and TAPDP-Keto^*^ in the S_0_ and S_1_ states, respectively.

**Table 1:** The absorption maxima (nm) of TAPHP and TAPDP in experimental and theoretical calculation.

	**Exp^a^**	**B3LYP**	**CAM-B3LYP**	**PBE0**	**M06-2X**	**PW6B95**
TAPHP. abs	450/295	495/368	392/306	477/354	401/304	475/309
TAPDP. abs	309	319	293	345	297	344

a * Maximal absorption peak in experiment*.

## Results and Discussion

### Geometrics

The two optimized geometric configurations (keto and enol) of TAPHP and TAPDP are shown in Figure 1B. To make the following description clearer, we labeled the atoms associated with HBs (O_1_, H_1_, O_2_, and N_1_). The relevant bond parameters are collected in Table 2. By comparing the HB parameters of TAPHP's enol form of S_0_ and S_1_ states, one can observe that the O_1_-H_1_ bond length is elongated by 0.019 Å (0.991 Å→1.01 Å), the distance of H_1_^**…**^O_2_ is shortened by 0.093 Å (1.607 Å→1.541 Å), and the bond angle of O_1_-H_1_^**…**^O_2_ is increased by 4.9° (149.6°→154.5°). Obviously, the strength of the O_1_-H_1_^**…**^O_2_ is strengthened in the S_1_ state, which is conducive to proton transfer. Optimization calculation shows that the keto form of TAPHP only forms in S_1_ state. In other words, TAPHP only performs ESIPT, rather than the ground state intramolecular proton transfer (GSIPT).

**Table 2:** Bond parameters of TAPHP-Enol, TAPHP-Keto, TAPDP-Enol, and TAPDP-Keto forms in the S_0_ and S_1_ states.

**Electronic state**	**TAPHP-Enol**		**TAPHP-Keto**		**TAPDP- Enol**		**TAPDP- Keto**	
	**S_**0**_**	**S_**1**_**	**S_**0**_**	**S_**1**_**	**S_**0**_**	**S_**1**_**	**S_**0**_**	**S_**1**_**
O_1_-H_1_	0.991	1.01	–	1.441	0.983	0.990	1.717	1.877
H_1_-O_2_	1.607	1.514	–	1.035	–	–	–	–
H_1_-N_1_	–	–	–	–	1.747	1.693	1.034	1.020
δ(O_1_-H_1_-O_2_)	149.6	154.5	-	157.4	–	–	–	–
δ(O_1_-H_1_- N_1_)	–	–	–	–	150.0	149.5	132.0	129.2

Moreover, HB interaction is found in TAPDP. By comparing the HB parameters of TAPDP's enol form of S_0_ and S_1_ states, we can find that the O_1_-H_1_ bond length is elongated by 0.007 Å (0.983→0.990 Å), the distance of H_1_^**…**^N_1_ is shortened by 0.054 Å (1.747 →1.693 Å), while the bond angle of O_1_-H_1_^**…**^N_1_ changes little. These changes indicate that O_1_-H_1_^**…**^N_1_ is enhanced in the S_1_ state. The keto form of TAPDP has been obtained by optimizing calculation. By comparing the HB parameters of TAPDP's keto form of S_0_ and S_1_ states, the distance of O_1_^**…**^H_1_ is shown to be elongated by 0.160 Å (1.717→1.877 Å), the bond length of H_1_-N_1_ is reduced by 0.014 Å (1.034→1.020 Å), and the bond angle of O_1_^**…**^H_1_-N_1_ is decreased by 0.8° (132.0→129.2°). Such variation can clearly show that O_1_^**…**^H_1_-N_1_ is stronger in the S_0_ state, which means the reverse proton transfer is more likely to occur in the S_0_ state.

### IR Vibration Analysis

**Figure 2:**
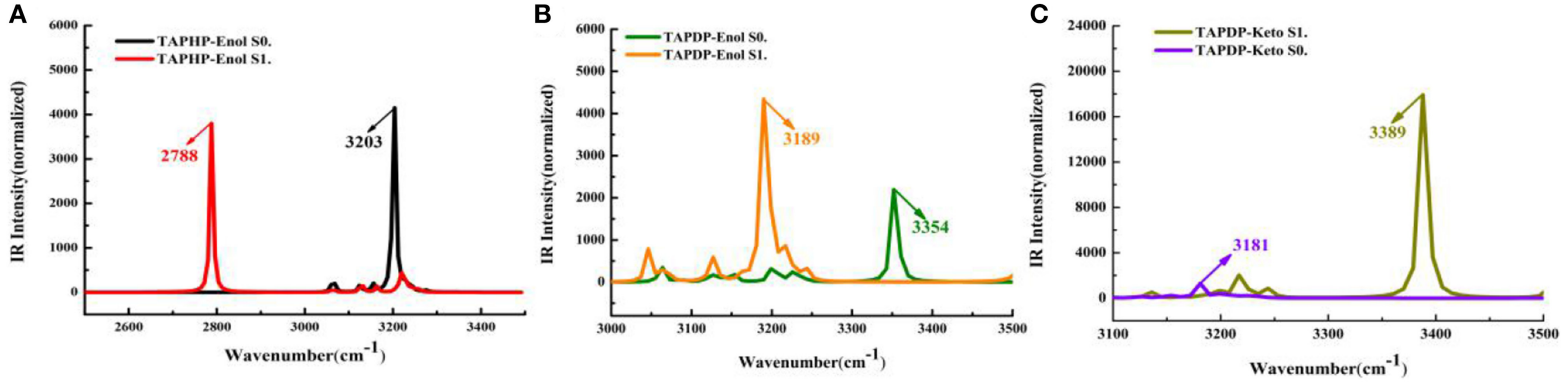
IR spectra of TAPHP and TAPDP. **(A)** TAPHP, O_1_-H_1_; **(B)** TAPDP, O_1_-H_1_; **(C)** TAPDP, H_1_-N_1_.

IR vibration spectra is a useful tool for analyzing hydrogen bonds. The IR spectra of O_1_-H_1_ and H_1_-N_1_ related to hydrogen bonds in TAPHP and TAPDP are displayed in a, b and c of Figure 2, respectively. It can be seen clearly from this figure that the vibration frequencies of O_1_-H_1_ stretching in TAPHP's enol form show the red-shift of 415 cm^−1^ (3,203→2,788 cm^−1^) from S_0_ to S_1_ states. The red shift of IR spectra indicates the enhancement of hydrogen bond, whereas the blue shift indicates the weakening of hydrogen bond. Obviously, the O_1_-H_1_^**…**^O_2_ of TAPHP's enol form is strengthened in S_1_ state.

Similarly, the stretching vibration frequencies of O_1_-H_1_ in TAPDP's enol form also show a red shift of 165 cm^−1^ (3,354→3,189 cm^−1^) from S_0_ to S_1_ states. This means that the O_1_-H_1_^**…**^N_1_ in TAPDP's enol form is enhanced in S_1_ state. However, the stretching vibration frequencies of H_1_-N_1_ in TAPDP's keto form reveal a blue shift of 208 cm^−1^ (3,181→3,389 cm^−1^) from S_0_ to S_1_ states. The conclusion that the O_1_^**…**^H_1_-N_1_ in TAPDP's keto form is stronger in S_0_ state is confirmed. Such conclusion is consistent with the above analysis of bond parameters.

### Hole-Electron Analysis

**Figure 3:**
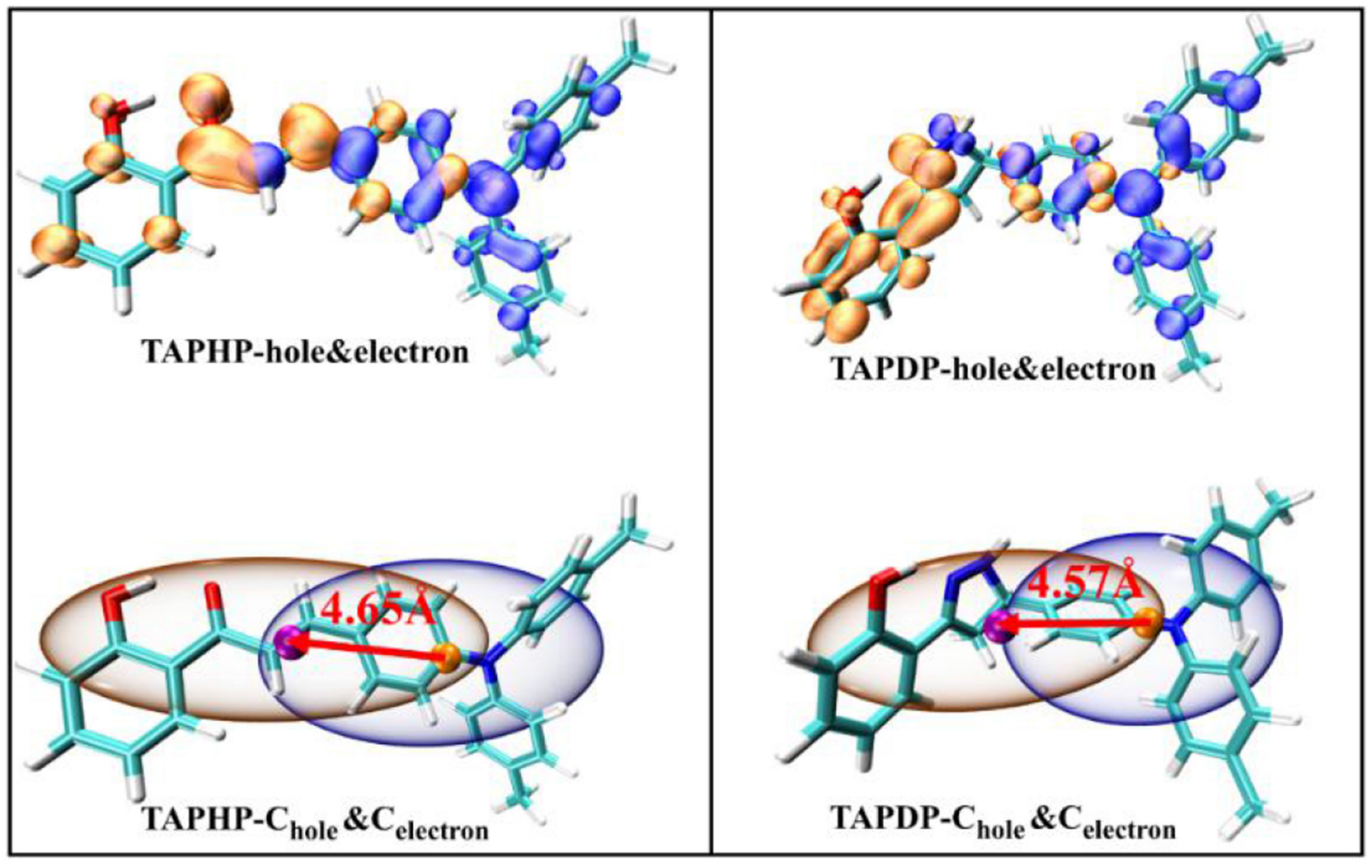
Hole-electron distribution and the C_hole_-C_ele_ diagrams of TAPHP and TAPDP.

Hole-electron analysis is an intuitive way of graphically examining electron excitation characteristics. The hole-electron distribution and the C_hole_-C_ele_ diagrams drawn by the Multiwfn program (Gao et al., 2019) are shown in Figure 3. The orange isosurfaces represents the electron distribution and the blue isosurfaces represents the hole distribution.

The C_hole_-C_ele_ diagram smoothes out the complex isosurfaces by erasing the details of hole and electron distribution. At the same time, Bahers et al. (2011). proposed a series of indexes including the D_CT_ index, which are adapted by Lu (2018) in the Multiwfn. The relevant indexes (*D, S*_r_, *H*, and *t*) are collected in Table 3. The four defined indexes and C_hole_-C_ele_ can be expressed by the formula:

(1) $$\begin{array}{rcl} D_{X} & = & \left|X_{ele}-X_{hole}\right| \end{array}$$

(2) $$\begin{array}{rcl} D_{Y} & = & \left|Y_{ele}-Y_{hole}\right| \end{array}$$

(3) $$\begin{array}{rcl} D_{Z} & = & \left|Z_{ele}-Z_{hole}\right| \end{array}$$

(4) $$\begin{array}{rcl} D\;\text{index} & = & \sqrt{{\left(D_{X}\right)}^{2}+{\left(D_{Y}\right)}^{2}+{\left(D_{Z}\right)}^{2}} \end{array}$$

(5) $$H_{\lambda }=\left(\sigma_{ele}{}_{,\lambda }+\sigma_{hole,\lambda }\right)/2\;\lambda \;=\left\{x,y,z\right\}$$

(6) $$\begin{array}{rcl} H_{CT} & = & \left|\text{H}\cdot \mu_{\text{CT}}\right| \end{array}$$

(7) $$H\text{ index}=\left(\left|\sigma_{ele}\right|+\left|\sigma_{hole}\right|\right)/2$$

(8) $$\begin{array}{rcl} t\;\text{index} & = & D\;\text{index}-H_{CT} \end{array}$$

(9) $$\begin{array}{rcl} S_{r}\;\text{index} & = & \int S_{r}\left(r\right)d\text{r}\equiv \int \sqrt{\rho^{hole}\left(r\right)\rho^{ele}\left(r\right)}\;d\text{r} \end{array}$$

where *X, Y, Z* (formulas 1, 2, 3) refer to the centroid coordinates of holes and electrons, respectively. The *D* index represents the distance between the centroid of the hole and the electron, which are 4.65 and 4.57 Å for TAPHP and TAPDP, respectively. The distance between the centroid of the hole and the electron is very large, which is obviously the result of the charge transfer excitation. And it can be seen from the hole-electron distribution map that electrons are transferred from triphenylamine to ketone. That is to say, both TAPHP and TAPDP undergo ICT instead of the experimental literature description that only TAPHP can occur. Both holes and electrons can be defined as σ (formula 5), and its three components of *x, y*, and *z* correspond to the root mean square deviation (RMSD) of holes or electrons distributed in the *x, y*, and *z* directions, reflecting the breadth of the distribution of holes and electrons. Therefore, the *H* index can reflect the overall average distribution breadth of electrons and holes. The *t* index measures the separation of holes and electrons. The *t* index > 0 implies that the separation of holes and electrons is sufficient due to charge excitation. In addition, *S*_r_ index indicates the overlap degree of holes and electrons.

**Table 3:** Indexes related to hole-electron distribution in TAPHP and TAPDP.

	***D*(Å)**	***S*_**r**_**	***H*(Å)**	***t*(Å)**
TAPHP	4.65	0.58	3.79	1.31
TAPDP	4.57	0.57	3.81	1.41

### Absorption and Emission Spectra

**Figure 4:**
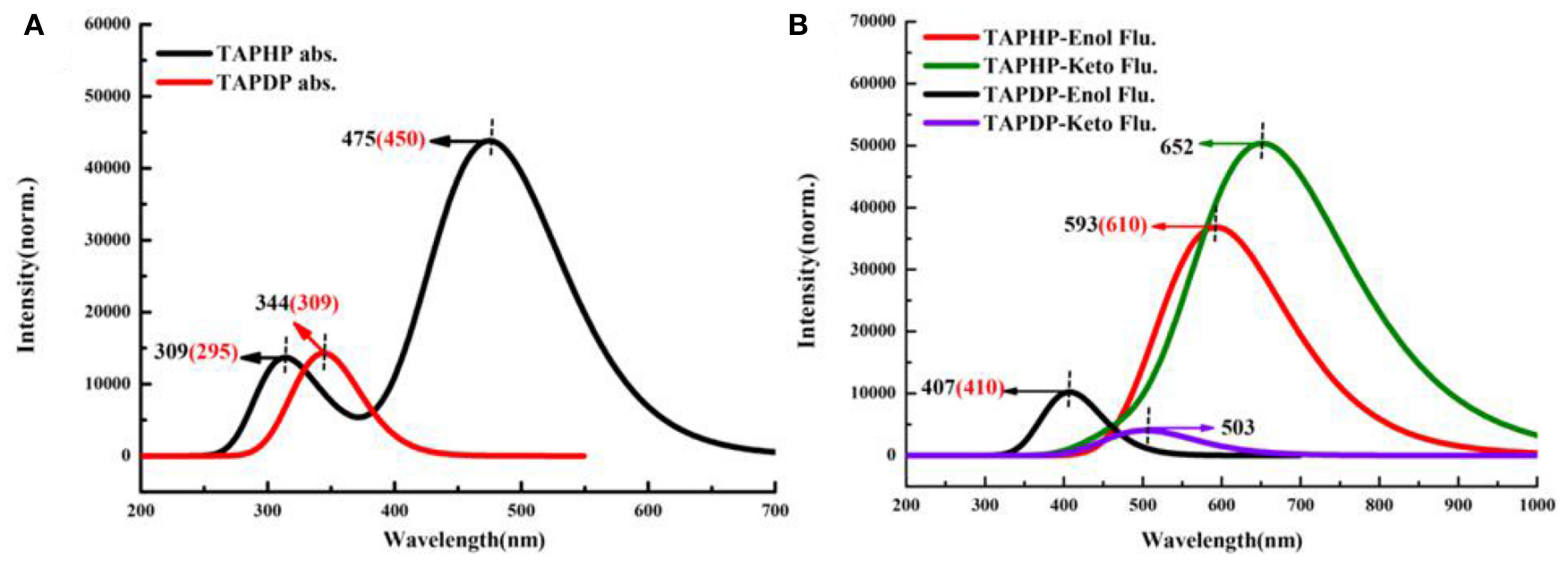
Electronic spectra of TAPHP and TAPDP in DMSO. **(A)** Absorption spectra. **(B)** Fluorescence spectra.

In order to more vividly illustrate the rationality of our calculation method, the electronic spectra of TAPHP and TAPDP are drawn in Figure 4. The calculated absorption peaks of TAPHP and TAPDP are 309, 475, and 344 nm, respectively, which coincide with the experimental values (295, 450, and 309 nm). The calculated results indicate that both the TAPHP and TAPDP are ESIPT fluorophores with double fluorescence emission, but only their single fluorescence peaks can be found in the experimental data. The reason that the fluorescence of TAPDP at 503 nm has not been experimentally observed can be considered to be that its intensity is too weak. We attribute the peak at 610 nm obtained in the experiment to the emission peak in the enol form of TAPHP, because the following PECs analysis indicates that TAPHP-Enol^*^ is stable in the S_1_ state. The fluorescence peak movement of TAPDP relative to TAPHP affords available sensing mechanism for the specific recognition of hydrazine.

### Topologocal Analysis

**Figure 5:**
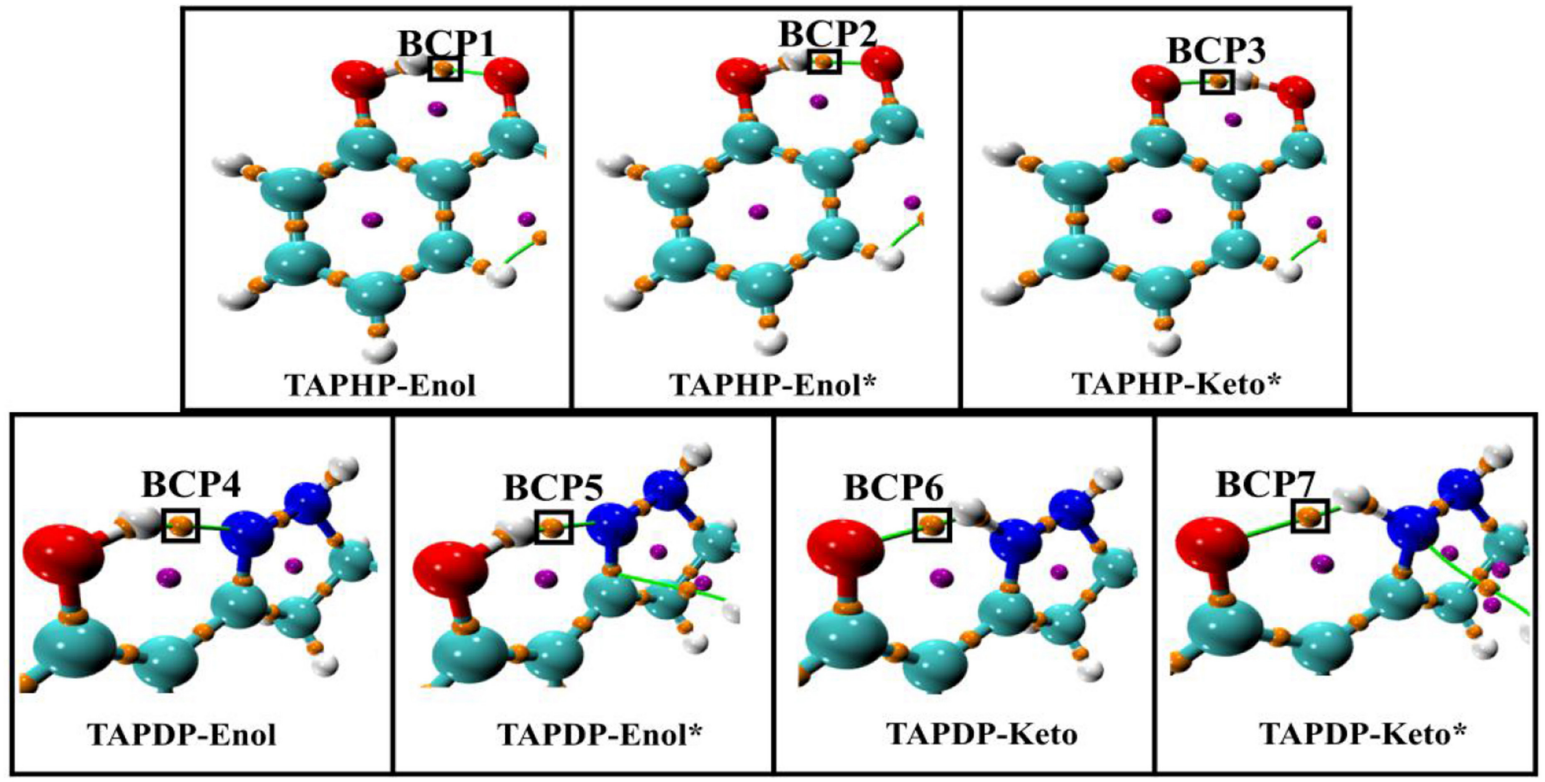
Topological analysis diagrams of TAPHP and TAPDP including bond paths and bond critical points.

Atoms-In-Molecule (AIM) theory, which describes bonding in molecules, is a reliable tool for characterizing HB interactions. The topological properties of electron density [ρ(r)] can be intuitively expressed in the Lewis structure of molecules by using the AIM method. According to Bader's theory (Bader and Essén, 1984), critical points and bond paths in equilibrium are both the necessary and sufficient conditions to specify the interaction (HB interaction) between two atoms. Topological analysis diagrams of geometric configurations related to HBs in TAPHP and TAPDP are displayed in Figure 5. Fortunately, there are bond critical points (BCPs) and bond paths between the two atoms associated with HBs in all configurations. This is a visual representation of the presence of HBs in the molecule. We call the BCPs of each configurations at different states as BCP*i* (*i* = 1,….7). The indicators (shown in Table 4) of BCPs are the focus of our attention because they are the key to the strength of the interaction. For homogeneous interactions, the higher the ρ(r) and the more negative the density of potential energy [V(r)] at the BCPs, the stronger the interaction between the two atoms connected by the bond path. It can be found that absolute values of ρ(r) and V(r) at BCPs follow the following relation: BCP2 > BCP1, BCP5 > BCP4, and BCP6 > BCP7. Meanwhile, the absolute values of hydrogen bond energy (E_HB_), Laplacian of electron density [∇ ^2^ρ(r)], kinetic energy density [G(r)] and total electron energy density [H(r)] also fit the rule well. The strength of HB can then be concluded to have the following relations: TAPHP-Enol^*^ > TAPHP-Enol, TAPDP-Enol^*^ > TAPDP-Enol, and TAPDP-Keto> TAPDP-Keto^*^. In addition, the indicators such as ρ(r) at BCP3 are also relatively high, which means that a strong HB favoring reverse proton transfer is formed in TAPHP -Keto^*^.

**Table 4:** Obtained parameters of BCPs in the TAPHP and TAPDP.

	**BCP1**	**BCP2**	**BCP3**	**BCP4**	**BCP5**	**BCP6**	**BCP7**
ρ(r) (a.u.)	0.0608	0.0767	0.0938	0.0461	0.0512	0.0488	0.0341
V(r) (a.u.)	−0.0690	−0.0910	−0.1133	−0.0437	−0.0504	−0.0508	−0.0310
E_HB(KJ/mol)_	90.58	119.46	148.73	57.38	66.16	66.69	40.70
∇^2^ρ(r)	0.1498	0.1552	0.1350	0.1166	0.1232	0.1410	0.1182
G(r)	0.0532	0.0649	0.0735	0.0364	0.0406	0.0430	0.0303
H(r)	−0.0158	−0.0261	−0.0398	−0.0073	−0.0098	−0.0078	−0.0007

### CVB Index

The CVB index obtained by using the topological analysis of electronic localization function (ELF) is a method proposed by Silvi et al. to research the strength of hydrogen bond (Fuster and Silvi, 2000). In the present work, hydrogen bond is represented as D-H^**…**^A, where D is the donor atom, and A is the acceptor atom. In the ELF basin analysis, this area is made up of the following ELF basins: V(D, H): valence basin formed by D atom and its bonding H atom, C(D) and C(A): the core basins of D and A atoms, V(A): valence bath of A atom. The CVB index is defined as:

(10) $$\begin{array}{r} \text{CVB index}=\text{ELF}\left(\text{C-V,D}\right)-\text{ELF}\left(\text{DH-A}\right), \end{array}$$

where ELF (C-V, D) is the core-valence bifurcation point value of D atom. ELF (DH-A) represents the bifurcation point value between V(D, H) and V(A). It has been verified that the more negative CVB index is, the stronger the HB is in general. The stronger the HB, the closer the distance between H atom and A atom, and the more covalent the interaction will be. Therefore, ELF (DH-A) is bound to become larger, resulting in a more negative CVB index. The CVB index of TAPHP and TAPDP in different states are displayed in Table 5. All the CVB indexes are negative, which indicate the existence of hydrogen bond interactions between the related atoms. Their relationship of absolute value of the CVB indexes are: TAPHP-Enol^*^ > TAPHP-Enol, TAPDP-Enol^*^ > TAPDP-Enol, and TAPDP-Keto> TAPDP- Keto^*^, which corresponds to the strength of hydrogen bond. It is worth to note that the CVB index of TAPHP-Keto in S_1_ state is the most negative, which demonstrates that O_1_^**…**^H_1_-O_2_ is a strong hydrogen bond.

**Table 5:** The CVB index of TAPHP and TAPDP in S_0_ and S_1_ states.

	**TAPHP-Enol**		**TAPHP- Keto**		**TAPDP- Enol**		**TAPDP- Keto**	
	**S_0_**	**S_1_**	**S_0_**	**S_1_**	**S_0_**	**S_1_**	**S_0_**	**S_1_**
ELF(C-V, D)	0.1033	0.1060	–	0.1121	0.0998	0.1004	0.1010	0.0983
ELF(DH-A)	0.2021	0.2682	–	0.3564	0.1719	0.1916	0.1601	0.1048
CVB index	−0.0988	−0.1622	–	−0.2443	−0.0721	−0.0911	−0.0592	−0.0066

### Independent Gradient Model

The method of investigating weak interactions by reduced the density gradient (RDG) is well-known. Recently, a new model (IGM) has been proposed that can precisely extract the characteristics of interactions in RDG diagrams (Lefebvre et al., 2017). The IGM provides a method for identifying and quantifying the gradient attenuation of net electron density. Its core is a descriptor (δg) that uniquely defines the region of interaction. The general way to calculate the density gradient [g(r)] of the pro-molecular is to sum over the density gradient of each atom, while the IGM type density gradient [g^IGM^(r)] is to sum over the absolute value of the density gradient of each atom. The difference between the two density gradients is called δg function, which is a three-dimensional real space function, can be written as,

(11) $$\begin{array}{r} g\left(r\right)=|\underset{i}{\sum }\nabla \rho_{i}\left(r\right)| \end{array}$$

(12) $$\begin{array}{r} g^{IGM}\left(r\right)=|\underset{i}{\sum }abs\left[\nabla \rho_{i}\left(r\right)\right]| \end{array}$$

(13) $$\begin{array}{r} \delta g\left(r\right)=g^{IGM}\left(r\right)-g\left(r\right) \end{array}$$

where *i* denotes the atom number, ∇_ρ_ the gradient vector, and *abs*(∇_ρ_) the absolute value of each component of the vector ∇_ρ_. The advantage of IGM is that it can examine the strength of interaction between each pair of atoms and quantify how much each atom affects the interaction between fragments.

**Figure 6:**
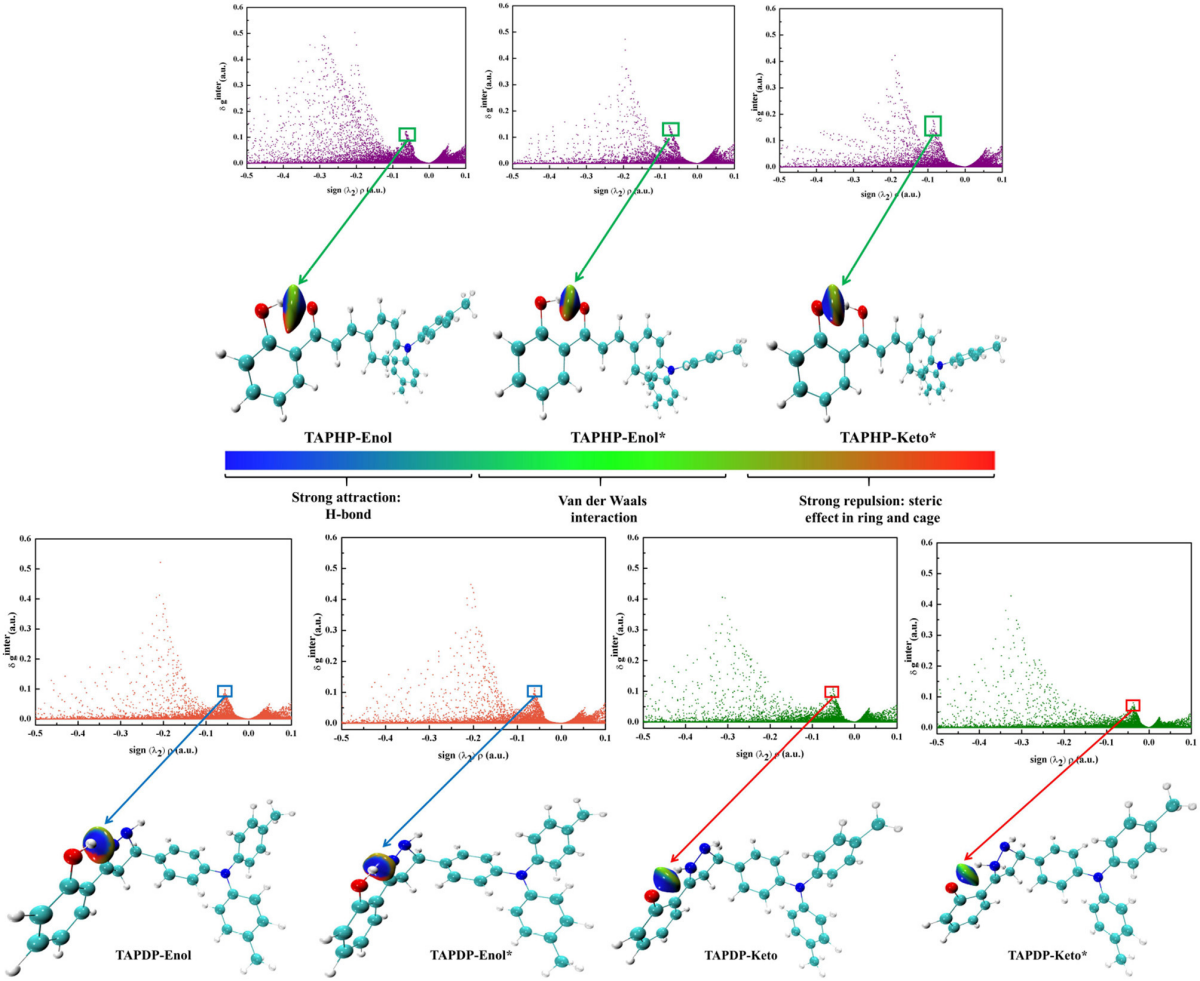
The scatter plots and gradient isosurfaces of TAPHP, TAPDP's enol form, and TAPDP's keto form.

We define the D-H of the HB interaction D-H^**…**^A as one fragment, A as another fragment. The scatter plots and isosurface maps, which can intuitively show the interaction, are drawn in Figure 6. The dark blue isosurfaces indicate that strong intramolecular HBs exist in TAPHP, TAPDP, and their isomers. The strength of the HBs can be shown by the spikes in the scatter diagrams. The peak value of O_1_-H_1_^**…**^O_2_ in S_1_ state is more negative than that in S_0_ state, which provide a strong proof for the enhancement of O_1_-H_1_^**…**^O_2_ in S_1_ state and the existence of O_1_^**…**^H_1_-O_2_ in S_1_ state as a strong hydrogen bond. For TAPDP, the peak value of O_1_-H_1_^**…**^N_1_ is more negative in S_1_ state than in S_0_ state, while the peak value of O_1_^**…**^H_1_-N_1_ has an opposite trend to that of O_1_-H_1_^**…**^N_1_. Obviously, O_1_-H_1_^**…**^N_1_ is stronger in the S_1_ state, while O_1_^**…**^H_1_-N_1_ is stronger in the S_0_ state. This further implies that proton transfer occurs in S_1_ state and reverse proton transfer occurs in S_0_ state.

### Potential Energy Curves

**Figure 7:**
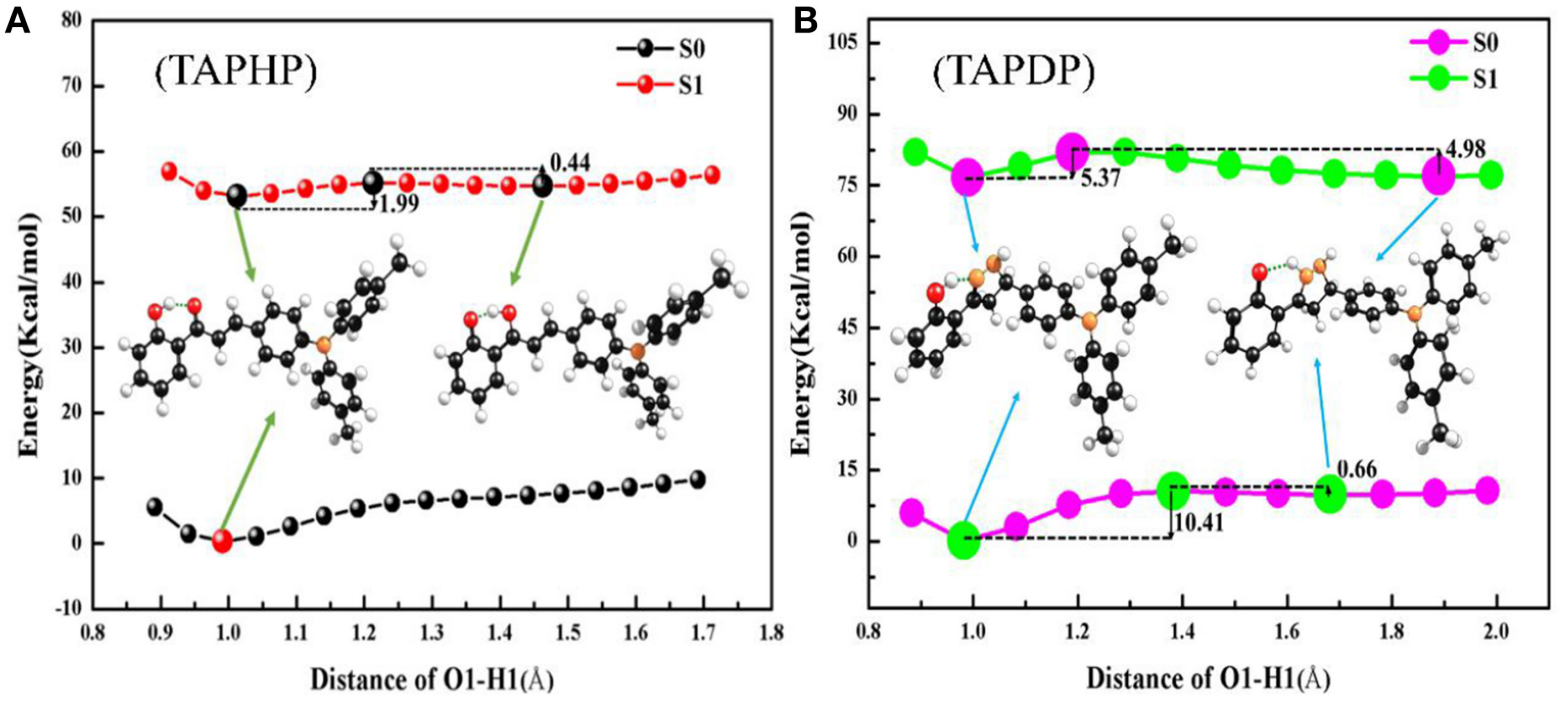
PECs and associated barriers of **(A)** TAPHP and **(B)** TAPDP.

To investigate the reaction path, the PECs of TAPHP and TAPDP are drawn in the Figure 7. As expected, the scans showed that the H proton moved along the reaction path near the O_1_ atom in TAPHP and the N_1_ atom in TAPDP. From this, we can speculate that proton transfer occurs in both TAPHP and TAPDP. According to the PECs of TAPHP, there is no local minimum corresponding to the stable keto form in S_0_ state but exists in S_1_ state, and there is only a small barrier (1.99 kcal/mol) from enol to keto form in S_1_ state. It is clear that the proton transfer of TAPHP can only occur in S_1_ state. In addition, the potential barrier (0.44 kcal/mol) needed for reverse proton transfer of TAPHP-Keto^*^ to form TAPHP-Enol^*^ is extremely small. Thus, TAPHP-Keto^*^ and TAPHP-Enol^*^ can coexist in S_1_ state. But the energy of TAPHP-Enol^*^ is less than the energy of TAPHP-Keto^*^, which means that TAPHP-Enol^*^ is most stable in S_1_ state. Thus, the dynamic process of TAPHP can be expressed as follows: TAPHP-Enol is firstly excited to S_1_ state (TAPHP-Enol^*^) by absorbing photons. Because of the small potential barrier, TAPHP-Enol^*^ will form the TAPHP-Keto^*^ by proton transfer. Then reverse proton transfer of TAPHP-Keto^*^ can easily occur due to the small reverse barrier and form the TAPHP-Enol^*^ isomer. Finally, the TAPHP-Enol^*^ performs a radiation transition back to the S_0_ state (TAPHP-Enol). This loop process reasonably explains why the emission peak of TAPHP's keto form was not observed in the experiment. However, TAPDP is quite different. It can be seen that the PECs have four stable points both in S_0_ and S_1_ states, namely, TAPDP–Enol, TAPDP-Keto, TAPDP-Enol^*^, and TAPDP-Keto^*^. The barrier to be crossed in S_0_ state is 10.41 kcal/mol, while the barrier in S_1_ state is only 5.37 kcal/mol. Obviously, ESIPT of TAPDP is easier to occur than GSIPT. The barriers that TAPDP needs to cross for reverse proton transfer in S_0_ and S_1_ states are 0.66 and 4.98 kcal/mol, respectively. Small barriers also make it possible for TAPDP to perform reverse proton transfer. The circulation system of TAPDP undergoing proton transfer should be: TAPDP-Enol→TAPDP-Enol^*^→TAPDP-Keto^*^→TAPDP-Keto →TAPDP-Enol.

### Analysis of Sensing Mechanisms

**Figure 8:**
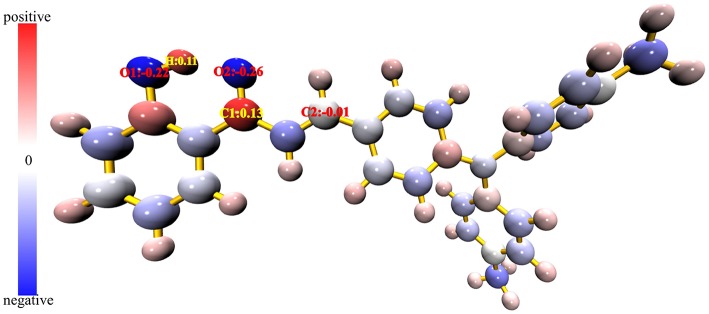
Hirshfeld atomic charge plotted in atomic coloring, the atomic charge corresponds to the blue-white-red color change from minimum to maximum.

It is known that hydrazine is more nucleophilic than hydroxylamine. The more positive the atomic charge, the easier it is to attract nucleophiles to react. It is therefore possible to verify the reaction site by calculating the atomic charge. The Hirshfeld atomic charge of the probe TAPHP is shown atomically colored in Figure 8. The size of the atomic charge increases in the order of blue, white and red. It can be seen that the atomic charge of C_1_ is the most positive, so C_1_ should be the site that most easily attracts the nucleophilic amino group. While the other amino group of hydrazine should react with C_2_ with an atomic charge close to 0. This is consistent with the reaction sites predicted experimentally. In order to verify the preference of the probe TAPHP for hydrazine, we calculated the binding energy of TAPHP for hydrazine and hydroxylamine, which are 4.59 and 3.56 kcal/mol, respectively. Therefore, the larger binding energy is one of the reasons why TAPHP shows excellent selectivity to hydrazine.

**Figure 9:**
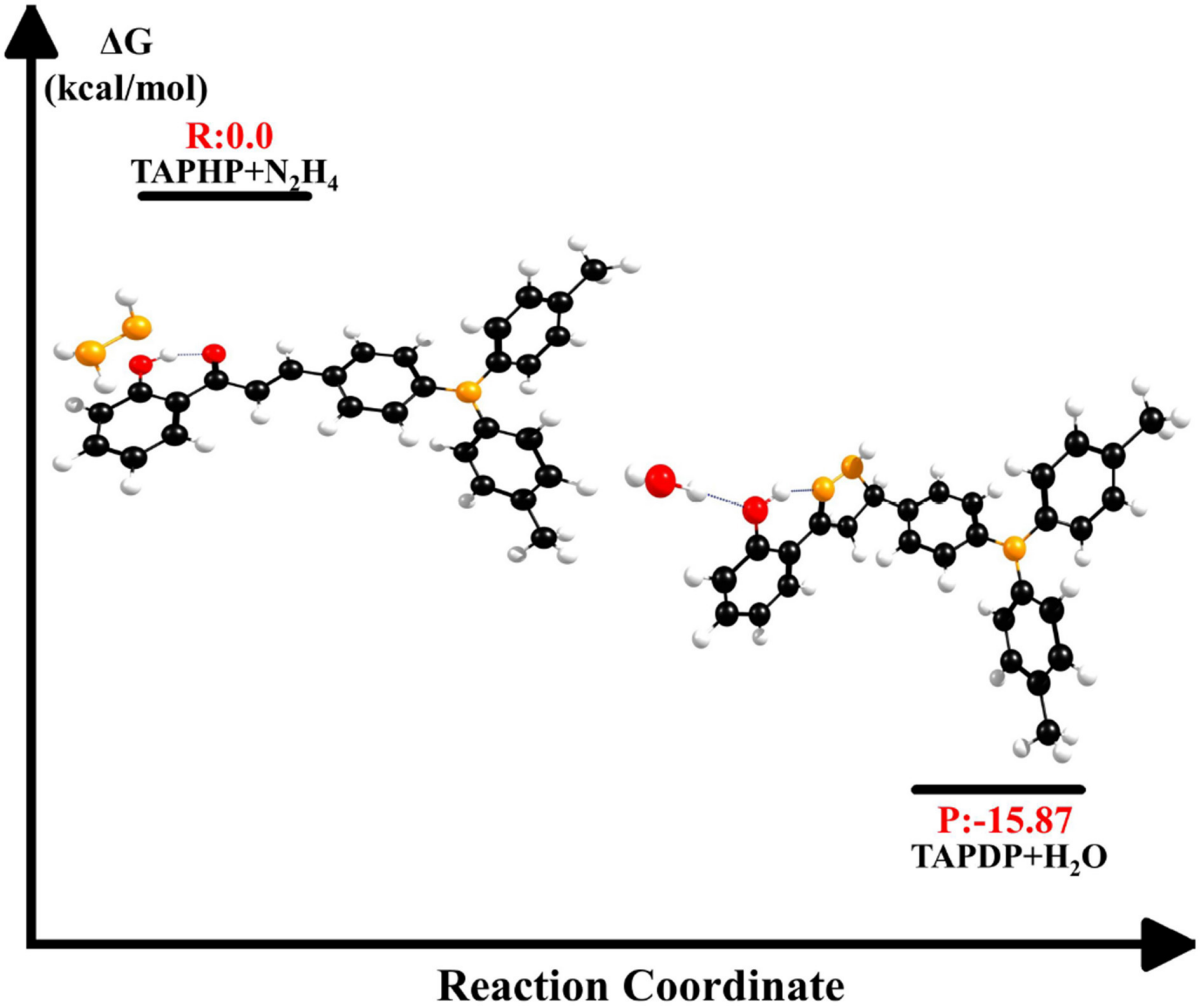
Free energy of reactants (TAPHP+N_2_H_4_) and products (TAPDP+H_2_O).

The energy changes in biochemical reactions that occur during biological oxidation can be described by thermodynamic free energy changes. As shown in Figure 9, the free energy of the products is −15.87 kcal/mol relative to the free energy of the reactants. The negative value of ΔG indicates that the reaction between TAPHP and hydrazine is an exothermic reaction, which can proceed spontaneously.

**Figure 10:**
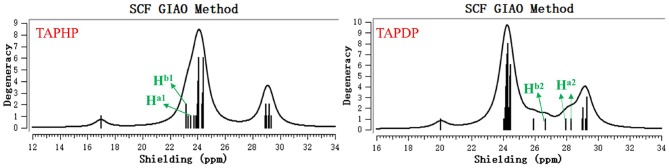
The ^1^H NMR spectra of TAPHP and TAPDP.

The ^1^H NMR spectra of TAPHP and TAPDP were obtained with tetramethylsilane (TMS) as reference material. As shown in Figure 10, the magnetic shield values of the two proton signals (H^a1^ and H^b1^) on the TAPHP were 23.18 and 23.53 ppm, and the NMR chemical shifts are 8.32 and 7.98 ppm, taking TMS as the standard. However, the peaks of TAPDP's signal at 23.18 and 23.53 ppm completely disappeared, and three new proton signals appeared, 26.68(4.82), 28.30(3.21), and 27.97(3.53) ppm, respectively. The data we obtained is consistent with the ^1^H NMR spectra measured experimentally. As a result, the configuration of the product TAPDP is affirmed. TAPHP's detecting mechanism for hydrazine are confirmed again.

## Conclusions

The ESIPT and ICT properties of the TAPHP and the TAPDP are researched using DFT and TDDFT methods. Analysis of the PECs can conclude that both TAPHP and TAPDP can have ESIPT instead of GSIPT. The bond parameters and IR vibrational spectra confirm the enhancement mechanism of hydrogen bond in the S_1_ state. The same conclusion is obtained by using the visualized isosurface maps equipped with IGM. Meanwhile, topological analysis based on AIM theory and CVB index are also applied to characterize the strength of hydrogen bonds. Hole-electron analysis suggests that both TAPHP and TAPDP undergo charge excitation, rather than only TAPHP as described in experimental literature, that is, ICT is inevitable. In addition, the calculated electronic spectra coincide with the experimental results. The fluorescence of TAPDP at 503 nm was not observed experimentally, which could be considered as the result of its weak intensity. Negative free energy difference implies a spontaneous exothermic reaction from TAPHP to TAPDP. Furthermore, TAPHP can indeed recognize hydrazine specifically by the movement of the fluorescence peaks. TAPHP's sensing mechanism for hydrazine is also characterized by atomic charge and ^1^H-NMR spectra.

## Data Availability Statement

The datasets analyzed in this manuscript are not publicly available. Requests to access the datasets should be directed to yzsong@sdnu.edu.cn.

## Author Contributions

SL, JL, and QL carried out the *ab initio* calculation. JF and LL analyzed the results. SL wrote the manuscript. CW and YS supervised this project.

### Conflict of Interest

The authors declare that the research was conducted in the absence of any commercial or financial relationships that could be construed as a potential conflict of interest.
